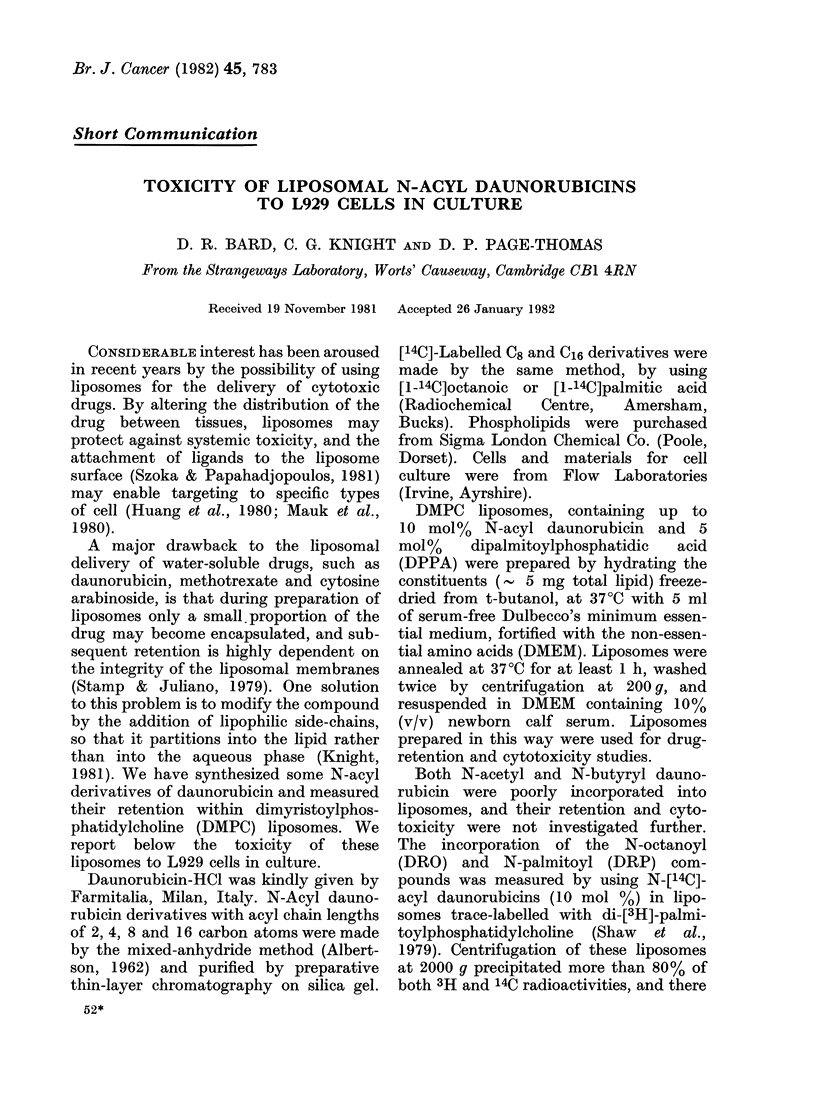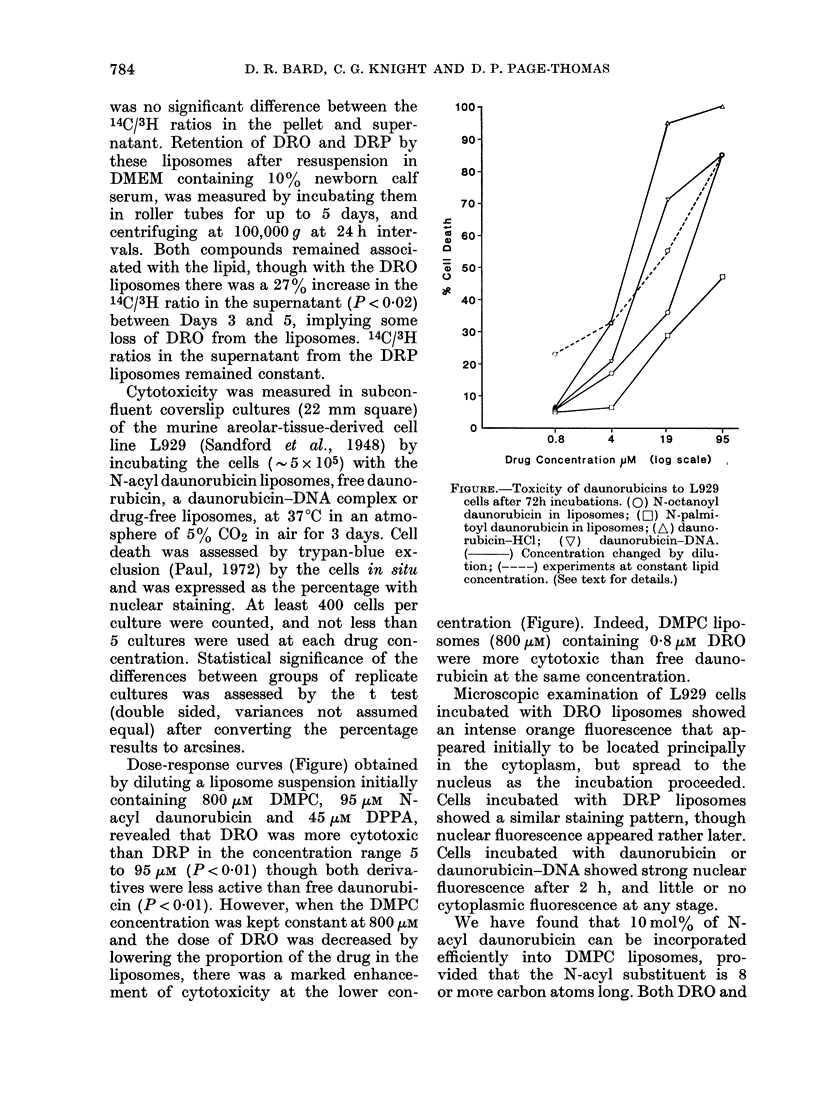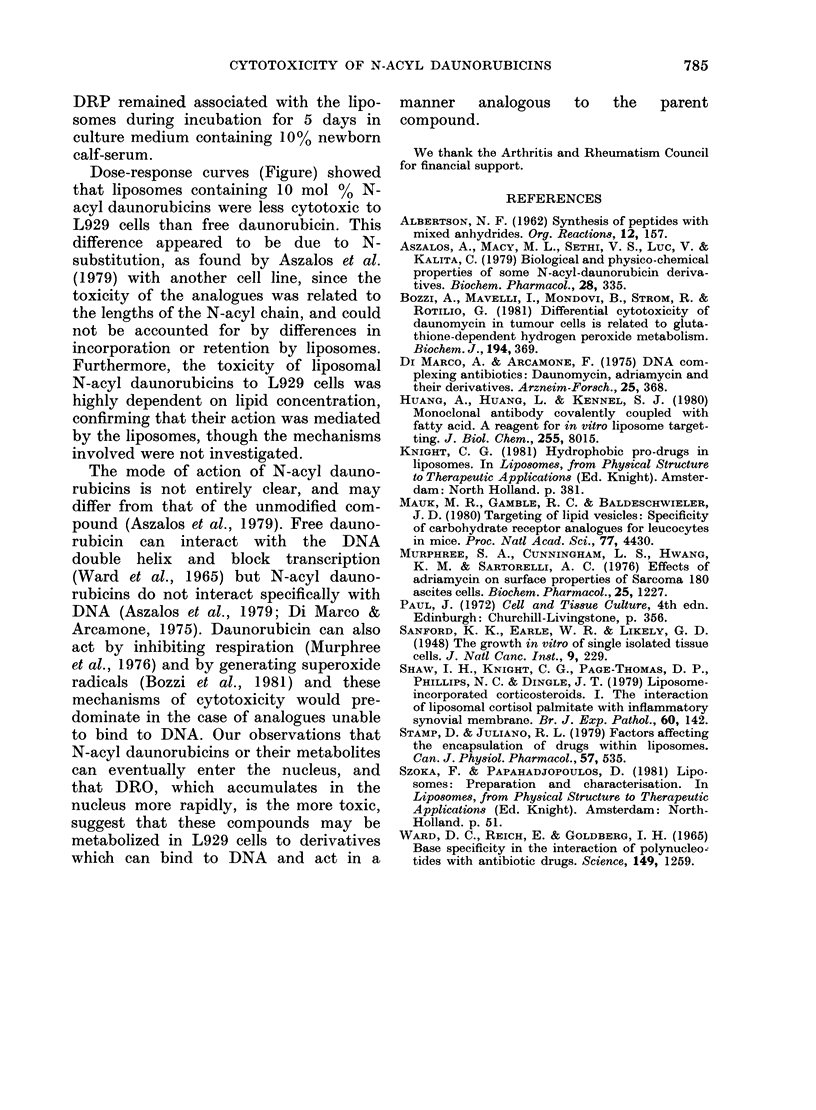# Toxicity of liposomal N-acyl daunorubicins to L929 cells in culture.

**DOI:** 10.1038/bjc.1982.121

**Published:** 1982-05

**Authors:** D. R. Bard, C. G. Knight, D. P. Page-Thomas


Br. J. Cancer (1982) **45**, 783

***Short Communication***

### TOXICITY OF LIPOSOMAL N-ACYL DAUNORUBICINS TO L929 CELLS IN CULTURE

D. R. BARD, C. G. KNIGHT AND D. P. PAGE-THOMAS

*From the Strangeways Laboratory, Worts' Causeway, Cambridge CB1 4RN*

Received 19 November 1981 Accepted 26 January 1982

CONSIDERABLE interest has been aroused in recent years by the possibility of using liposomes for the delivery of cytotoxic drugs. By altering the distribution of the drug between tissues, liposomes may protect against systemic toxicity, and the attachment of ligands to the liposome surface (Szoka & Papahadjopoulos, 1981) may enable targeting to specific types of cell (Huang *et al.*, 1980; Mauk *et al.*, 1980).

A major drawback to the liposomal delivery of water-soluble drugs, such as daunorubicin, methotrexate and cytosine arabinoside, is that during preparation of liposomes only a small proportion of the drug may become encapsulated, and subsequent retention is highly dependent on the integrity of the liposomal membranes (Stamp & Juliano, 1979). One solution to this problem is to modify the compound by the addition of lipophilic side-chains, so that it partitions into the lipid rather than into the aqueous phase (Knight, 1981). We have synthesized some N-acyl derivatives of daunorubicin and measured their retention within dimyristoylphosphatidylcholine (DMPC) liposomes. We report below the toxicity of these liposomes to L929 cells in culture.

Daunorubicin-HCl was kindly given by Farmitalia, Milan, Italy. N-Acyl daunorubicin derivatives with acyl chain lengths of 2, 4, 8 and 16 carbon atoms were made by the mixed-anhydride method (Albertson, 1962) and purified by preparative thin-layer chromatography on silica gel. [$^{14}$C]-Labelled $C_8$ and $C_{16}$ derivatives were made by the same method, by using [1-$^{14}$C]octanoic or [1-$^{14}$C]palmitic acid (Radiochemical Centre, Amersham, Bucks). Phospholipids were purchased from Sigma London Chemical Co. (Poole, Dorset). Cells and materials for cell culture were from Flow Laboratories (Irvine, Ayrshire).

DMPC liposomes, containing up to 10 mol% N-acyl daunorubicin and 5 mol% dipalmitoylphosphatidic acid (DPPA) were prepared by hydrating the constituents ($\sim$ 5 mg total lipid) freeze-dried from t-butanol, at 37°C with 5 ml of serum-free Dulbecco's minimum essential medium, fortified with the non-essential amino acids (DMEM). Liposomes were annealed at 37°C for at least 1 h, washed twice by centrifugation at 200 *g*, and resuspended in DMEM containing 10% (v/v) newborn calf serum. Liposomes prepared in this way were used for drug-retention and cytotoxicity studies.

Both N-acetyl and N-butyryl daunorubicin were poorly incorporated into liposomes, and their retention and cytotoxicity were not investigated further. The incorporation of the N-octanoyl (DRO) and N-palmitoyl (DRP) compounds was measured by using N-[$^{14}$C]-acyl daunorubicins (10 mol %) in liposomes trace-labelled with di-[$^{3}$H]-palmitoylphosphatidylcholine (Shaw *et al.*, 1979). Centrifugation of these liposomes at 2000 *g* precipitated more than 80% of both $^{3}$H and $^{14}$C radioactivities, and there was no significant difference between the $^{14}C/^{3}H$ ratios in the pellet and supernatant. Retention of DRO and DRP by these liposomes after resuspension in DMEM containing 10% newborn calf serum, was measured by incubating them in roller tubes for up to 5 days, and centrifuging at 100,000 $g$ at 24 h intervals. Both compounds remained associated with the lipid, though with the DRO liposomes there was a 27% increase in the $^{14}C/^{3}H$ ratio in the supernatant ($P < 0{\cdot}02$) between Days 3 and 5, implying some loss of DRO from the liposomes. $^{14}C/^{3}H$ ratios in the supernatant from the DRP liposomes remained constant.

Cytotoxicity was measured in subconfluent coverslip cultures (22 mm square) of the murine areolar-tissue-derived cell line L929 (Sandford *et al.*, 1948) by incubating the cells ($\sim 5 \times 10^5$) with the N-acyl daunorubicin liposomes, free daunorubicin, a daunorubicin–DNA complex or drug-free liposomes, at 37°C in an atmosphere of 5% $CO_2$ in air for 3 days. Cell death was assessed by trypan-blue exclusion (Paul, 1972) by the cells *in situ* and was expressed as the percentage with nuclear staining. At least 400 cells per culture were counted, and not less than 5 cultures were used at each drug concentration. Statistical significance of the differences between groups of replicate cultures was assessed by the t test (double sided, variances not assumed equal) after converting the percentage results to arcsines.

Dose-response curves (Figure) obtained by diluting a liposome suspension initially containing 800 $\mu$M DMPC, 95 $\mu$M N-acyl daunorubicin and 45 $\mu$M DPPA, revealed that DRO was more cytotoxic than DRP in the concentration range 5 to 95 $\mu$M ($P < 0{\cdot}01$) though both derivatives were less active than free daunorubicin ($P < 0{\cdot}01$). However, when the DMPC concentration was kept constant at 800 $\mu$M and the dose of DRO was decreased by lowering the proportion of the drug in the liposomes, there was a marked enhancement of cytotoxicity at the lower concentration (Figure). Indeed, DMPC liposomes (800 $\mu$M) containing 0·8 $\mu$M DRO were more cytotoxic than free daunorubicin at the same concentration.

FIGURE.—Toxicity of daunorubicins to L929 cells after 72h incubations. (○) N-octanoyl daunorubicin in liposomes; (□) N-palmitoyl daunorubicin in liposomes; (△) daunorubicin-HCl; (▽) daunorubicin–DNA. (———) Concentration changed by dilution; (----) experiments at constant lipid concentration. (See text for details.)

Microscopic examination of L929 cells incubated with DRO liposomes showed an intense orange fluorescence that appeared initially to be located principally in the cytoplasm, but spread to the nucleus as the incubation proceeded. Cells incubated with DRP liposomes showed a similar staining pattern, though nuclear fluorescence appeared rather later. Cells incubated with daunorubicin or daunorubicin–DNA showed strong nuclear fluorescence after 2 h, and little or no cytoplasmic fluorescence at any stage.

We have found that 10 mol% of N-acyl daunorubicin can be incorporated efficiently into DMPC liposomes, provided that the N-acyl substituent is 8 or more carbon atoms long. Both DRO and

DRP remained associated with the liposomes during incubation for 5 days in culture medium containing 10% newborn calf-serum.

Dose-response curves (Figure) showed that liposomes containing 10 mol % N-acyl daunorubicins were less cytotoxic to L929 cells than free daunorubicin. This difference appeared to be due to N-substitution, as found by Aszalos *et al.* (1979) with another cell line, since the toxicity of the analogues was related to the lengths of the N-acyl chain, and could not be accounted for by differences in incorporation or retention by liposomes. Furthermore, the toxicity of liposomal N-acyl daunorubicins to L929 cells was highly dependent on lipid concentration, confirming that their action was mediated by the liposomes, though the mechanisms involved were not investigated.

The mode of action of N-acyl daunorubicins is not entirely clear, and may differ from that of the unmodified compound (Aszalos *et al.*, 1979). Free daunorubicin can interact with the DNA double helix and block transcription (Ward *et al.*, 1965) but N-acyl daunorubicins do not interact specifically with DNA (Aszalos *et al.*, 1979; Di Marco & Arcamone, 1975). Daunorubicin can also act by inhibiting respiration (Murphree *et al.*, 1976) and by generating superoxide radicals (Bozzi *et al.*, 1981) and these mechanisms of cytotoxicity would predominate in the case of analogues unable to bind to DNA. Our observations that N-acyl daunorubicins or their metabolites can eventually enter the nucleus, and that DRO, which accumulates in the nucleus more rapidly, is the more toxic, suggest that these compounds may be metabolized in L929 cells to derivatives which can bind to DNA and act in a manner analogous to the parent compound.

We thank the Arthritis and Rheumatism Council for financial support.

### REFERENCES

Albertson, N. F. (1962) Synthesis of peptides with mixed anhydrides. *Org. Reactions*, **12**, 157.

Aszalos, A., Macy, M. L., Sethi, V. S., Luc, V. & Kalita, C. (1979) Biological and physico-chemical properties of some N-acyl-daunorubicin derivatives. *Biochem. Pharmacol.*, **28**, 335.

Bozzi, A., Mavelli, I., Mondovi, B., Strom, R. & Rotilio, G. (1981) Differential cytotoxicity of daunomycin in tumour cells is related to glutathione-dependent hydrogen peroxide metabolism. *Biochem. J.*, **194**, 369.

Di Marco, A. & Arcamone, F. (1975) DNA complexing antibiotics: Daunomycin, adriamycin and their derivatives. *Arzneim-Forsch.*, **25**, 368.

Huang, A., Huang, L. & Kennel, S. J. (1980) Monoclonal antibody covalently coupled with fatty acid. A reagent for *in vitro* liposome targetting. *J. Biol. Chem.*, **255**, 8015.

Knight, C. G. (1981) Hydrophobic pro-drugs in liposomes. In *Liposomes, from Physical Structure to Therapeutic Applications* (Ed. Knight). Amsterdam: North Holland. p. 381.

Mauk, M. R., Gamble, R. C. & Baldeschwieler, J. D. (1980) Targeting of lipid vesicles: Specificity of carbohydrate receptor analogues for leucocytes in mice. *Proc. Natl Acad. Sci.*, **77**, 4430.

Murphree, S. A., Cunningham, L. S., Hwang, K. M. & Sartorelli, A. C. (1976) Effects of adriamycin on surface properties of Sarcoma 180 ascites cells. *Biochem. Pharmacol.*, **25**, 1227.

Paul, J. (1972) *Cell and Tissue Culture*, 4th edn. Edinburgh: Churchill-Livingstone, p. 356.

Sanford, K. K., Earle, W. R. & Likely, G. D. (1948) The growth *in vitro* of single isolated tissue cells. *J. Natl Canc. Inst.*, **9**, 229.

Shaw, I. H., Knight, C. G., Page-Thomas, D. P., Phillips, N. C. & Dingle, J. T. (1979) Liposome-incorporated corticosteroids. I. The interaction of liposomal cortisol palmitate with inflammatory synovial membrane. *Br. J. Exp. Pathol.*, **60**, 142.

Stamp, D. & Juliano, R. L. (1979) Factors affecting the encapsulation of drugs within liposomes. *Can. J. Physiol. Pharmacol.*, **57**, 535.

Szoka, F. & Papahadjopoulos, D. (1981) Liposomes: Preparation and characterisation. In *Liposomes, from Physical Structure to Therapeutic Applications* (Ed. Knight). Amsterdam: North-Holland. p. 51.

Ward, D. C., Reich, E. & Goldberg, I. H. (1965) Base specificity in the interaction of polynucleotides with antibiotic drugs. *Science*, **149**, 1259.